# The Role of Influencers and Echo Chambers in the Diffusion of Vaccine Misinformation: Opinion Mining in a Taiwanese Online Community

**DOI:** 10.2196/57951

**Published:** 2025-08-18

**Authors:** Jason Dean-Chen Yin, Tzu-Chin Wu, Chia-Yun Chen, Fen Lin, Xiaohui Wang

**Affiliations:** 1 Li Ka Shing Faculty of Medicine, School of Public Health, University of Hong Kong, Hong Kong, China; 2 College of Public Health, Institute of Health Behaviors and Community Sciences, National Taiwan University, Taiwan, Taiwan; 3 Department of Media and Communication, City University of Hong Kong, Room 5086, Creative Media Center, Hong Kong, China, 852 34428691

**Keywords:** misinformation, vaccine, online community, influencer, echo chamber, Taiwan

## Abstract

**Background:**

Prevalence and spread of misinformation are a concern for the exacerbation of vaccine hesitancy and a resulting reduction in vaccine intent. However, few studies have focused on how vaccine misinformation diffuses online, who is responsible for the diffusion, and the mechanisms by which that happens. In addition, researchers have rarely investigated this in non-Western contexts particularly vulnerable to misinformation.

**Objective:**

This study aims to identify COVID-19 vaccine misinformation, map its diffusion, and identify the effect of echo chamber users on misinformation diffusion on a Taiwanese online forum.

**Methods:**

The study uses data from a popular forum in Taiwan called PTT. A crawler scraped all threads on the most popular subforum from January 2021 until December 2022. Vaccine-related threads were identified through keyword searching (n=5818). Types of misinformation, including misleading, disinformation, conspiracy, propaganda, and fabricated content, were coded by 2 researchers. Polarity was proposed as a proxy for measuring an individual’s level of involvement in the echo chamber, one of the mechanisms responsible for the viral misinformation on social media. Factors related to information diffusion, including misinformation type and polarity, were then assessed with negative binomial regression.

**Results:**

Of 5818 threads, 3830 (65.8%) were identified as true information, and 1601 (27.5%) contained misinformation, yielding 5431 boards for analysis. Misinformation content did not vary much from other contexts. Propaganda-related information was most likely to be reposted (relative risk: 2.07; *P*<.001) when comparing to true information. However, the more polarized a user was, the less likely his or her content was to be reposted (relative risk: 0.22; *P*<.001). By removing the nodes with a high level of indegree, outdegree, and betweenness centrality, we found that the core network and the entire network demonstrated a decreasing trend in average polarity score, which showed that influential users contributed to the polarization in misinformation consumption.

**Conclusions:**

Although the forum exhibits a resilience to echo chambering, active users and brokers contribute significantly to the polarization of the community, particularly through propaganda-style misinformation. This popularity of propaganda-style misinformation may be linked to the political nature of the forum, where public opinion follows “elite cues” on issues, as observed in the United States. The work in this study corroborates this finding and contributes a data point in a non-Western context. To manage the echo chambering of misinformation, more effort can be put into moderating these users to prevent polarization and the spread of misinformation to prevent growing vaccine hesitancy.

## Introduction

### Background

As individuals increasingly turn to social media as a primary source of information, the prevalence and spread of unverified or misinformed scientific claims is concerning [1]. Social media users gravitate toward information that validates their belief systems, forming echo chambers that validate their shared narrative [2]. These echo chambers can be the launching pads for misinformation that goes viral [3]. Worse, they can influence opinions on issues of public concern, such as vaccination [4].

As early as 2001, studies identified the rise of vaccine hesitancy online [5,6] and cataloged the techniques in antivaccination misinformation transmission [7,8]. These studies paralleled the early days of the World Wide Web and focused solely on analyzing web pages. An early study by Zimmerman et al [6] found that misrepresentation (twisting of content) was a method for conveying vaccine misinformation. Kata [8] elaborated on misrepresentation in her study in 2010 on childhood vaccination. In her study, misinformation, a sort of misrepresentation, was a main antivaccination theme that arose in classification. Under the misinformation umbrella, she found that using outdated sources; misrepresenting facts; self-referencing to “experts”; not referencing statistics or citations; and making unsupported statements were all ways of passing misinformation. The use of negative tones is also a method of strengthening antivaccination methods [9].

As misinformation became more prevalent on social media, exacerbated by the terming of “fake news” and a global pandemic, more research was conducted on clarifying the definition of misinformation and classifying the different kinds of misinformation. The term “misinformation” is often used interchangeably with related concepts such as spam, rumors, fake news, and disinformation. In this study, we use “misinformation” as an umbrella term to encompass all false or inaccurate information disseminated through social media. Under these umbrellas, there are different types of misinformation. Some information can be fabricated content, while some can be manipulated to be clickbait. Some can be satire or parody being passed off as true, and some can be propaganda [10]. Information can be passed off as true via sponsors and be partially true or totally false. New modes of producing synthetic media through artificial intelligence such as “deep fakes” distort reality by passing as true. Although there is nuance between the different types of misinformation, all types are distinct from true information, which is not deliberately fabricated with malicious intent and does not contain false (scientific) information.

There are several studies on classifying types of vaccine misinformation. Wu et al [11], in their study on general misinformation in social media, tentatively organized misinformation into nonexclusive categories such as unintentionally and intentionally spread misinformation, urban legend, fake news, unverified information, rumor, crowdturfing, spam, and trolling. Zhao et al*’s* [12] study does not deviate far from this. They identified that misinformation could include conspiracies; concerns about vaccine safety and efficacy; a flat rejection of all vaccines; morality (including religion and human experimenting); and a violation of civil liberties. Classification studies also identified new modes of transmission. Basch et al [13] studied the types of misinformation on TikTok, a medium mixing audiovisual and textual cues, and found that parodying or the overemphasis of false consequences of available vaccines were all methods of strengthening an antivaccination narrative.

In addition to modes of transmission and classification, some studies have focused on linking misinformation to vaccination intent, although these are sparse. One study used a questionnaire to identify that COVID-19 vaccine hesitancy mediated the relationship between vaccine knowledge and vaccination intention, with misinformation on vaccines being associated with higher vaccine hesitancy. The same study also claimed that most respondents were exposed to COVID-19 misinformation [14]. Another study conducted a randomized controlled trial, finding that exposure to recent misinformation induced a decline in intent of vaccination. This is especially true when the misinformation is transmitted as misrepresenting facts of a scientific nature [15].

In the studies on vaccine misinformation, there are unexplored avenues. Few studies have examined how vaccine misinformation diffuses online, both in time and in quantity. Diffusion is a communication process whereby information is communicated over time “among the members of a social system” [16]. Often, this process is measured in terms of depth, breadth, and speed [17,18]. Diffusion depth refers to the length of the longest transmission chain, breadth refers to the capacity to generate offspring posts, and speed refers to the efficiency of the information diffusion process [19]. Understanding the diffusion process between true information and misinformation is important because, should approaches such as psychological inoculation [20] or other vaccination community strategies work [21,22], understanding the scale of spread and when to intervene diminishes the spread.

In addition, no studies have studied key users’ roles in spreading vaccine misinformation. This can be broken down into 2 symbiotic perspectives. One perspective is looking at how individuals’ engagements online may be polarizing or divisive. Previous research on polarity focused on this mostly in politics, using different metrics of measurement for polarity. One study assessed political polarization using interactional, positional, and affective polarity across various platforms to find that polarization is platform dependent [23]. Another study looked at affective polarization against the feminist cause following protests [24]. In the vaccination literature, polarity mostly focuses on differences in vaccine sentiment [4,25,26], aligning it with affective polarization. However, there are a paucity of studies analyzing polarity in engagement with misinformation. This is important because the deliberate exclusion of true perspectives on vaccination may affect the downstream intention to vaccinate. Echo chambers that form around misinformation are thus one avenue worth exploring. Previous research has found that echo chambers are related closely to misinformation diffusion [27] and that misinformation disseminated by echo chambers spread more virally than misinformation not distributed by them [28].

The second perspective is identifying who the key users are in this process. One can see how the polarity in the endorsement of misinformation can be exponentially more problematic when the users doing the exclusion are both laypeople [29] and central in the network. Two studies found that information diffused by “brokers”—those who have high betweenness centrality—affects the final size of information cascades [30,31]. For health information, the diffusion size of posts by the US Centers for Disease Control and Prevention (CDC) were related to broker involvement [32]. More work can thus be done on understanding how key users aid in spreading vaccine misinformation through the deliberate exclusion of “true news,” thereby enhancing its dissemination.

The consequence of having these key users spread information lies in the fact that individual beliefs are shaped by their immediate social network, both online and offline [33]. These worlds feed back to each other, entrenching belief. Scholars have found that dissemination of misinformation is driven by social reinforcement in an individual’s digital circles [34]. If a person’s network consists of mostly rumormongers, that person in turn likely propagates the same misinformation. This propagation often spills into the offline space, influencing judgment through reinforcing the legitimacy of the information online [35], eventually creating fissures along sociodemographic lines, further polarizing the offline world [36]. Should this process occur for vaccine information, it would exacerbate the issue of vaccine hesitancy and have negative implications for vaccination. Few studies have examined the upstream part of this process, including in non-Western contexts [36]. This study focuses on vaccine misinformation on a local forum in Taiwan (PTT) from 3 aspects: identifying the types of misinformation, describing how it diffuses relative to regular news, and assessing how influential users fall into chambers of misinformation, affecting its spread.

### Research Questions

This study uses the same PTT data to address the following questions about misinformation. First, we will determine what types of misinformation are present in a Taiwanese online community. This identification and categorization archives how misinformation topics differ to other contexts considering the sociopolitical context of Taiwan. Second, the study will determine how misinformation cascades differ from true information by dichotomizing the topics, comparing their breadth [19]. Finally, we will assess to what extent the echo chamber phenomenon exists in the diffusion of misinformation and how influential users, as a proxy for understanding key spreaders of misinformation, affect the echo chamber in the network. The findings of this study can inform how Taiwan can combat growing misinformation in its diverse online ecology.

## Methods

### Data Procurement

PTT is a terminal-based bulletin board system developed in Taiwan in 1995. Functioning like an internet forum, PTT is often termed Taiwan’s “Reddit” and is one of the most active forums in Taiwan. From July 2022 to July 2023, the average number of users per day was 56,000 [37]. The web-based version of PTT is structured into different boards*,* which are equivalent to thematic groups, on a forum. Within each board, threads or topics can be started. On each thread, the poster’s metadata such as the username, time of post, and IP address are available for scraping. In addition, within the threads there is a comment section for which the corresponding metadata is also available.

The data for this study were collected from the “Gossiping” board on PTT using a web scraper developed in Python to extract HTML data. The scraping targeted posts made between January 2021 and December 2022. We filtered the dataset using the Chinese term for “vaccine” to ensure we captured vaccine-related threads. The language of all collected data was Mandarin Chinese, the primary language used on PTT. The “Gossiping” board was specifically chosen due to its high activity levels and its reputation for users engaging in a wide range of discussions, including those related to public health, politics, and societal issues. This board is particularly active during times of political or social crises, making it a rich source for analyzing vaccine misinformation during the COVID-19 pandemic. Other thematic boards were excluded to maintain focus on a general-purpose discussion forum where misinformation may more organically spread across a wider audience. In total, 5818 boards were pulled.

### Identifying Misinformation

The coding process for classifying vaccine misinformation followed an established framework based on existing literature. We used a broad classification scheme (Table 1) derived from studies on misinformation in digital spaces, which categorize misinformation into types such as conspiracies, fabricated content, fake news, and political propaganda. The classification of posts into misinformation categories was conducted in Mandarin by 2 trained coders, who worked independently to classify an initial sample of 500 posts. The initial codebook was developed using an inductive coding approach, where new categories emerged from the data during this pilot phase. The coders reached a high level of intercoder reliability, with a Cohen κ of 0.91, indicating strong agreement. Discrepancies were resolved through discussion, and the finalized codebook was used to classify the remaining 5318 posts.

**Table 1.** Labeling scheme for true information and false information.

Category and label	Classification criteria
**True information**	
Factual news	Reporting of news events or facts without interpretation or analysis
Scientifically accurate analytical content	Interpreting or analyzing facts or data to provide deeper understanding
**Misinformation**	
Misleading information	False or inaccurate information, regardless of intention
Disinformation	Deliberately created and shared with the intent to mislead or deceive
Propaganda	Biased or misleading information used to promote a political cause or push a certain point of view
Fabricated content	Entirely false content designed to deceive and mislead (create a fake news source, fake quotes, or nonexistent events)
Conspiracy	Belief or explanation that something is a result of a secret plot by a group or organization
Mixed misinformation	Two or more misinformation categories
Religious beliefs	Any discussion of religion in relation to vaccination
Unrelated	Boards containing “vaccine” keyword but unrelated to vaccine information or misinformation

Any disagreements among coders were resolved through discussion among themselves. The coders informed the principal investigator of these discrepancies, who further approved or reassessed their decision, informing the coders. As a standard reference, we consolidated a library of misinformation on COVID-19 vaccines in Taiwan from three fact-checking organizations in Taiwan: MyGoPen [38], Cofacts [39], and Taiwan FactCheck Center [40]. These organizations, led by civil society movements combatting misinformation, consolidated potentially misinformative news on a variety of topics, including those related to COVID-19. Boards related to the COVID-19 vaccine were filtered by using “vaccine” as a keyword in Chinese. In the event of disagreements on classification, the vaccine-related misinformation on these sites was used as a final check. Following the initial calibration, the remaining 5318 boards were split into 2 datasets (n=2659) and independently coded. Any questions or concerns were brought up to all coders and the principal investigator; the final classification was determined by a majority vote or mutual agreement. For comparing diffusion of misinformation and users’ polarized engagement with misinformation, the categories in Table 1 are further collapsed into “true” and “false” information. Posts unrelated to vaccines or involving purely religious content were excluded from the final analysis. Thematic examples of excluded posts include those focused on general prayer requests, religious teachings not tied to vaccination, or unrelated social and political discussions. For example, posts discussing the afterlife or religious rituals were categorized as unrelated, as they did not directly engage with the vaccine misinformation discourse.

For subsequent analyses, we wanted to remove users who occasionally shared misinformation and focus on users who shared misinformation frequently (ie, we distilled a “core network” by extracting users with over 5 posts; n≥5, n=number of posts). This is also consistent with previous studies that used multiple filtering techniques to remove randomness in a large network [41,42]. To justify the choice of cutoff, robustness tests were conducted, as shown in Multimedia Appendix 1. In short, different threshold values (from 1 to 10) and the average polarity scores of the selected core network were calculated to see any significant changes. Although the average polarity increased as the cutoff increased, the trend of decreasing polarity by cutting out key users was the same across all values. To balance between cutting off too many or too few nodes for a forum, the choice of n≥5 was made.

### Diffusion of Misinformation

The diffusion of information is traditionally measured in 3 ways: breadth, depth, and speed. These 3 aspects follow previously defined measurements on diffusion on social media platforms [18]. However, given the structure of the PTT forum, depth is unascertainable. The reason for this is because, in PTT, replies of replied posts always refer to the original post, and not the replied post. This metadata obscures the length of the diffusion chain. Compare this to platforms like X (formerly Twitter) that link the diffusion chain through explicitly stating a “retweet” of a “retweet” is “retweeted” from the “retweet,” and not the original tweet. Due to the structure in PTT, breadth will be used to capture the size of the information diffusion. Breadth is the number of first-degree child nodes that repost it. If we denote a message as *m* and the set of first-degree child nodes as *N*(*m*), the breadth *B*(*m*) is equivalent to |*N*(*m*)|.

In addition, a negative binomial regression model will estimate the predictors of breadth. A negative binomial regression is used since overdispersion is expected for the distribution of breadth of information diffusion. To build the model, the categorical misinformation types will be input as categorical variables, with “true information” as the baseline. Besides, the polarity score generated in the next section will be used. Polarity is included because it is possible that users who tend to be extreme on either spectrum are likely to have more engagement in the network. In addition, 2 control variables will be used, including the word count of the post and the activeness of the users. Previous research has suggested that longer posts have a higher likelihood of being transmitted [43]. The activeness of a user is operationalized as the number of historical posts. Although activity on a media platform does not necessarily mean higher engagement [44], it is an important confounding factor to be included in the model. The regression model estimate is: log (*μ_i_*) = *β*_0_ + *β*_1_(*Misinformation*) + *β*_2_(*Disinformation*) + *β*_3_(*Propaganda*) + *β*_4_(*Fabricated*) + *β*_5_(*Propaganda*) + *β*_6_(*WordCount*) + *β*_7_(*UserActiveness*) + *β*_8_(*PolarityScore*) + *ε.*

where *μ_i_* is the count of the dependent variable for the *i*^*th*^ observation of breadth of misinformation spread. The log link function relates the mean of the response variable to the linear predictors. Variables are tested for multicollinearity prior to inclusion into the model. For a deeper analysis into the variable correlations, see Multimedia Appendix 2. Given that there is no offset term in the regression, the results of the negative binomial regression produce a relative risk (RR) indicating the increased risk of the outcome variable.

### Measuring Polarity

Polarization in public opinion refers to the extent to which the views of a population (in this case, support for true or false information) are extreme and distinctly divided [45]. In the context of this study, polarity represents the predominant commenting activity on either true or false information (ie, misinformation), suggesting that users are chambered in their engagement with certain types of information. This measure aligns with the “affective polarization” measures previously used in the politics [23,24] and vaccine sentiment literature; however, they are extended to this study to measure endorsement of either true or false information. Further, polarization can be measured at the individual level or aggregated at the community level. In this study, we measure individuals’ level of polarity, proposing 2 metrics for analysis.

The first is polarity measured by the difference in proportion of comments on true information and misinformation (“proportion polarity”). To measure this, we collected the commenting behavior for each node *v* in the network. With their commenting history, *C*(*v*), we calculated the number of comments on true and false information, denoted as *C_pos_*(*v*) and *C_neg_*(*v*), respectively. The proportions of positive and negative comments were then $P_{pos\left(v\right)}=\frac{C_{pos}(v)}{C(v)}$ and $P_{neg}\left(v\right)=\frac{C_{neg}\left(v\right)}{C(v)}$, respectively. We subtracted the proportion of negative comments from the proportion of positive comments to get the polarity score, π_*prop*_(*v*), per node using the equation π_*prop*_(*v*) = *P_pos_*(*v*) – *P_neg_*(*v*). The range of π_*prop*_(*v*) is –1 ≤ π_*prop*_(*v*) ≤ 1, with a score of −1 representing a polarity in commenting only on false information, 0 representing equal commenting on both, and 1 representing entirely commenting on true information.

**Figure 1.**
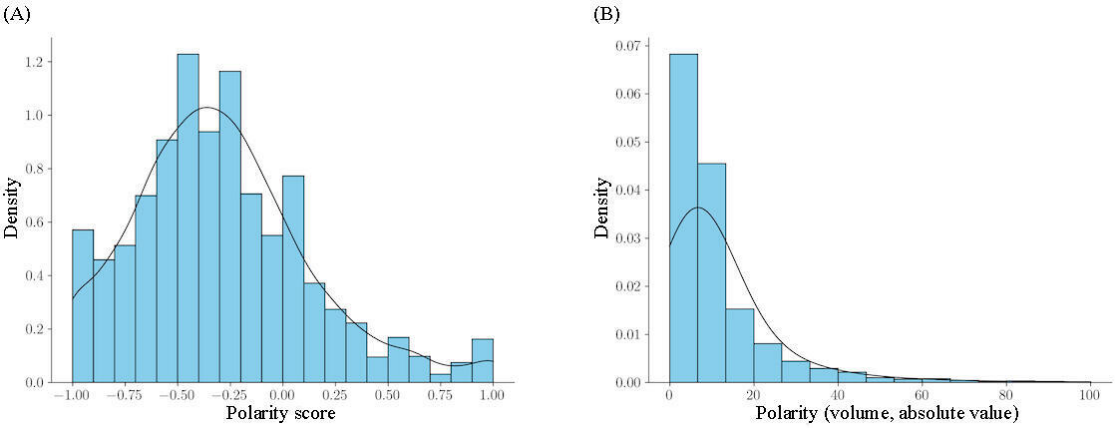
Distribution of polarity scores (n=2422) calculated by (A) proportion and (B) volume for the core network.

The second is polarity measured by the absolute value of the difference in volume of comments on true and false information (“volume polarity”). Measuring by absolute value of the difference removes the true-false dichotomy and allows a straightforward interpretation of any polarity in the network, permitting a clearer interpretation of echo chambering. To calculate volume polarity, π_*vol*_(*v*), we took the absolute value of the difference of the number of negative to positive comments, |*C_neg_*(*v*) – *C_po_*_s_(*v*). The range of π_*vol*_(*v*) is then 0 ≤ π_*vol*_(*v*) < *inf*, with higher values indicating higher polarity. Figure 1 illustrates the distribution in polarity scores calculated by proportion (Figure 1A) and volume (Figure 1B) for the core network. For ease of interpretation in the negative binomial regression, volume polarity value is min-max normalized to create a value between 0 and 1 due to the expected heavier left skew. To preserve the weight of posting in information diffusion, we use the polarity by volume in our following analyses.

### Node-Level Predictors of Polarity

Influential users generally have disproportionate impact on the flow of network information, also shown in the previous section. Identifying them and their relation to polarity in the network is therefore an important step in diminishing the spread of misinformation. Measures of centrality are usually used to identify node importance in the network. We used 2 centrality metrics to identify influential nodes, with each representing a different concept of influence.

The first measure of centrality is degree centrality, which directly measures the number of connections a node has. The more connections a node has, the more central and influential it is within the network. In PTT, since forum data is directional, the indegree and outdegree of a node will be calculated for each node separately. Decomposing degrees into indegrees and outdegrees helps distinguish whether nodes on either polar end are more an authority (indegree) or a broadcaster (outdegree) of misinformation.

The second measure of centrality is betweenness centrality, which measure the extent to which a node lies on paths between other nodes; specifically, capturing the frequency with which a node appears on the shortest paths between pairs of nodes. Nodes with high betweenness centrality have control over information flow in the network, acting as “bridges” or “brokers” across the network [46]. These nodes are critical for the flow of information in the network because they function as “switches” for facilitating or inhibiting information flow. For the network, let σ*_st_* denote the total number of shortest paths from node *s* to node *t*, and σ*_st_(*v) denote the number of those paths that pass through a node *v*. The betweenness centrality *b*(*v*) can be defined as:


$$\sum_{s\neq v\neq t}\frac{\sigma_{st}\left(v\right)}{\sigma_{st}}$$

which sums over all pairs of nodes (*s,t*) in the network, excluding those for which the pair is *v*, and for each pair, calculates the fraction of shortest paths between *s* and *t* that pass through *v*. Said another way, it calculates the proportion of times *v* is a bridge along the shortest path between 2 other nodes.

To analyze how polarity in the network changes in response to these influencers in misinformation diffusion, we removed a subset of the percentage of top influential users—by increasing increments of 5%—and calculated and plotted the average absolute value polarity score in the network. The resulting graphs of changing polarity indicate the change in overall posting behavior on either true or misinformative threads by influential users.

### Ethical Considerations

This study involved the analysis of secondary data collected from publicly available social media platforms. The data were obtained from PTT, an open-access online forum, and all posts were publicly accessible at the time of data collection. No private or sensitive information was collected, and all user data were anonymized before analysis. The study did not involve any interaction with the individuals behind the social media accounts, and no identifiable information is included in the results. All findings are presented in aggregate to ensure anonymity. As the research only analyzed publicly available data and did not involve human subjects directly, formal ethics approval was not required according to institutional guidelines. Nonetheless, the study adhered to ethical principles of data privacy and responsible handling of social media data.

## Results

### Identified Misinformation

The results in Table 2 show the number of boards by each misinformation type in Table 1, in addition to an elaboration of several of the most common thread types. Out of 5818 threads, most threads (n=2227, 38.3%) involved netizens asking questions for further clarification on vaccines. The next most common was reporting of official news reports or press conference news (n=1603, 27.8%). For all threads containing misinformation, the most common was the “propaganda” type (n=858, 14.7%), indicating the relatively political nature of this forum. After disregarding the unrelated and religious threads, and recategorizing the “mixed*”* threads into the conspiracy category, there were 3830 true threads and 1601 threads containing misinformation, for a total of 5431 boards for analysis moving forward. For these boards, the number of comments and reposts for true and false information is illustrated in Table 3.

**Table 2.** Counts and examples of common misinformation types on PTT.

Label	Threads, n (%)	Common types found	Example
**True information (n=3830, 65.8%)**			
Factual news	1603 (27.6)	Official reports of vaccine side effects from government sites, often containing a “News” tag, with text fully reported. Most report official announcements or press conference news with no expression of opinion.	Title: “[News] Free registration now open! Vaccination booth stationed at the PX Mart near Christmas Land”
Scientifically accurate analytical content	2227 (38.3)	Nonbiased question-asking by “villagers”^a^ that do not carry any slant in content, or a tone that instigates comments.	“Recently, there was a debate on vaccination, which we only heard the opposite side to not vaccinate. Yes, there are always safety concerns for any medical technology, and it is not 100% preventive of reinfection, but are there other reasons?”
**Misinformation (n=1601, 27.5%**)			
Misleading information	452 (7.8)	Appears as a question (or comment) but has negative intentions of misleading the public (without understanding the origin of the intention).	“We are now allowing children to get Moderna vaccines, but the side effects are large. According to Murphy’s Law, even if the probability is low, it is still possible. If a child does die, after vaccination, who is responsible? … looking at a dazzling object, it’s just tin foil. Behind cute makeup is just powder; even polished nails have dark edges.”
Disinformation	97 (1.7)	Linking the vaccine as a direct cause of other diseases or ailments (death, cyborgism, myocarditis, balding, etc), and deriving these conclusions from personal experience.	“A lot of people are experiencing side effects from the vaccine, and if something happens, no one takes responsibility. Young people do not need vaccination since their ability to recover is incredibly strong, right? QQ”
Propaganda	858 (14.7)	Linking the reasons for vaccination to specific political attitudes. Often have the characteristic of replying to news posts, guided by personal opinions to specific political positions.	“The ‘Taliban’^b^ really is ahead internationally, even half of their supporters refuse to roll up their sleeves for Medigen [Taiwan’s domestic vaccine]. But maybe they can use it for a bath? The party can enjoy a good bath in their benevolent fluid”
Fabricated content	195 (3.4)	Making nonfactual statements or describing stories related to vaccines based on unwarranted assumptions. Often begins with statements such as “I dreamed that...” or “My friend did...” It is difficult to verify the level of fabrication through text.	“Recently, a female college went to her OBGYN after her Moderna injection. She said her menstruation came twice a month. Some people also said after BNT, the heart was uncomfortable, and took a few days to pass. Why are there still people taking the third dose of MRNA? What’s the gossip?”
Conspiracy	179 (3.1)	Linking vaccine administration or distribution with the hidden interests of the government and pharmaceutical companies. Mostly done along the lines of them achieving the “benefits” of controlling people if they control vaccine distribution.	“The original vaccines are developed using the original virus, but the virus is constantly mutating. Vaccine factories are not quick to develop new ones, and just encourage us to take booster doses. Isn’t this just being done to cheat us of our money? Just like Intel, slowly squeezing out toothpaste, then launching the next generation of products when they can no longer make money.”
Mixed misinformation	29 (0.5)	Most cases of 2 or more involved some analytical component, used in a propaganda way.	—^c^
Religious beliefs	23 (0.4)	Adding religious slogans or arguments after raising vaccine-related views. In some texts, elaborated arguments include the viewpoints of a “false world” or “unknown forces.” Could also be categorized as broadly conspiracy, with religious grounding.	“I believe [username] now. He said that vaccines are all planned to change human beings. Viruses are man-made and everything is planned. He said the heart used to be on the left side of the body, and now it’s in the center according to Google. I’m convinced. I do not want the vaccine. Do you believe [username]?”
Unrelated	155 (2.7)	—	—

a Villagers is a common term used on PTT that roughly translates to netizens.

b “Taliban” is a derogatory internet slang term that refers to the Democratic Progressive Party or “green” party in Taiwan. The Mandarin Chinese term substitutes the “li” in “Taliban” for the homophonic sound for “green.”

c Not applicable.

**Table 3.** Number of comments and reposts by true information and misinformation.

	Average number of comments	Average number of reposts
True information	74.01	0.40
**Misinformation**	52.63	0.20
Misleading information	52.63	0.20
Disinformation	75.16	0.16
Propaganda	66.63	0.39
Fabricated content	50.21	0.20
Conspiracy theories	51.41	0.26

Based on our coding and descriptive analysis of the different types of misinformation, we found that the narratives in Taiwan closely resemble those observed in Western contexts. One example is the conspiracy theory that vaccination is used as a means of control by governments or corporations [47,48]. Broadly speaking, these are symptoms of an overarching lack of trust in authorities on health, and part of the larger antiscience trend [48-52]. This point is potentially exacerbated in geopolitically tense contexts such as Taiwan. One such example was the many narratives of faulty quality control prevalent for the Pfizer BioNTech vaccine, since its distributor for Greater China, Fosun, was Shanghai-based, and vaccines were refused as a result. The frequent discussion about and linking of vaccine decisions and political parties (66.63 comments per board, 0.39 average reposts, Table 3) also corroborates this point, and it is also a trend found in the United States. As a side note, the United States and Taiwan share similar forms of government systems, as well as bipartisan polarization.

The following analyses use the core network. In the entire network, there were 23,004 unique users, with the average polarity (by volume) being 3.27. The core network is relatively smaller and more polarized than the entire network, with 6035 unique users with an average polarity of 8.70.

### Diffusion of Misinformation

Overall, the average word count of posts was 525.5 words while the median was 326.0, indicating a heavy right tail. The same trend was observed for the activeness of the user, with an average of 279.3 engagements—any posts or comments—compared to a median of 129.0. The polarity score of the core network is long-tail distributed, and it has an average of 38.2 but a median of 4 (Figure 1). For breadth, among threads identified containing true information, 608 (15.9%) were shared at least once. Conversely, 165 (10.3%) of misinformative threads were reposted. There is no statistical difference between the average repost counts across true information and misinformation, with means of 2.39 and 2.33 reposts for true information and misinformation, respectively (*t*=0.250; *P*=.803). The 95% confidence interval for the 2 values ranged from −2.2 to 29.1 for true information threads and −1.1 to 10.3 for misinformation threads.

Table 4 presents negative binomial regression results of predicting breadth of information diffusion. As can be seen, the RR of repost for propaganda information is double that of true information (RR=2.07; *P*<.001). The risk of reposting disinformation is half that of true information (RR=0.48; *P*=.001). Moreover, posts from more polarized users (RR=0.22; *P*<.001*)* do not arouse as much discussion, suggesting the forum is relatively averse to echo chambering in relation to misinformation.

**Table 4.** Predictors of breadth (n=2422).

Variables	Breadth		
	Exp(coef)	SE	*P* value
**Information type**			
True information (baseline)	N/A^a^		
Misleading information	1.10	0.085	.28
Disinformation	0.48	0.227	.001
Propaganda	2.07	0.063	<.001
Fabricate information	0.79	0.156	.12
Conspiracy theories	1.23	0.132	.12
Polarity of user	0.22	0.202	<.001
**Control variables**			
Word count (of post)	1.0002	0.25 × 10^–4^	<.001
User activeness (number of historical posts)	0.998	0.53 × 10^–4^	.001

a Not applicable.

### Node-Level Predictors of Polarity

**Figure 2.**
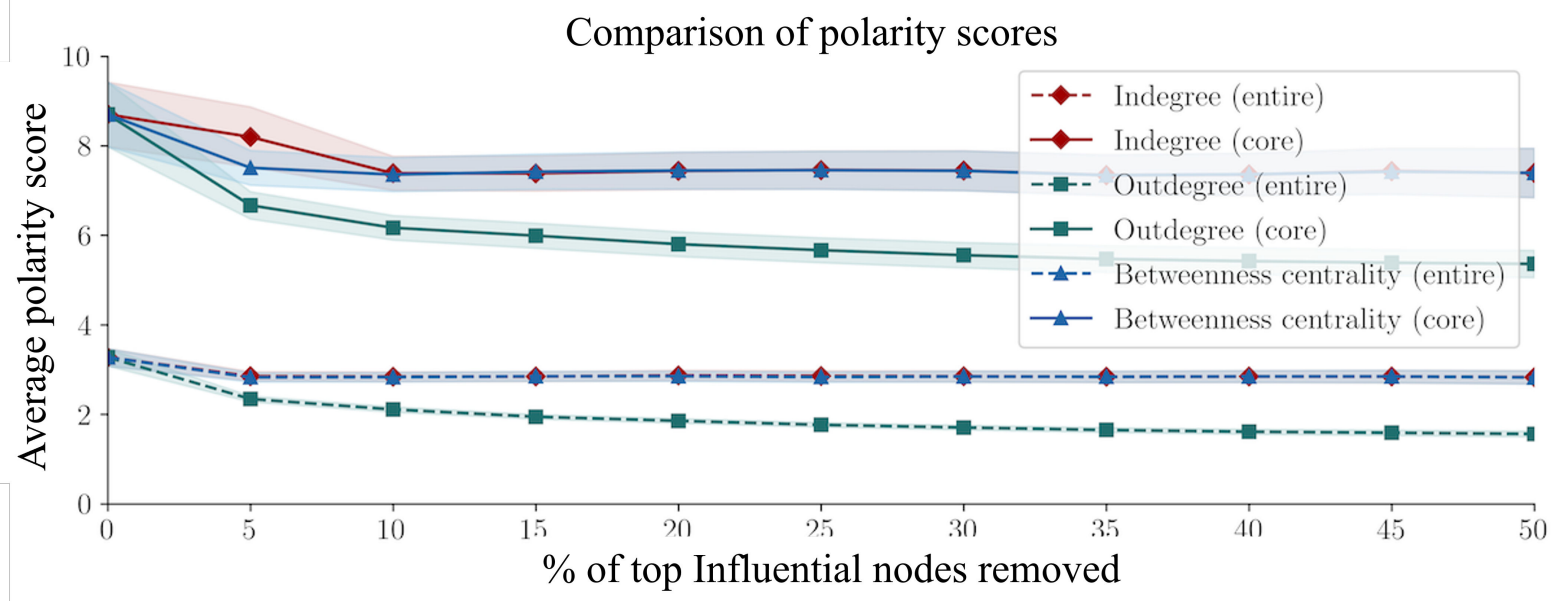
Impact of removing top percent of influential nodes on network (polarity score calculation: absolute value of volume).

We also examined whether removing influential users would contribute to decreasing polarization in the misinformation diffusion network. Figure 2 shows the changes of polarity scores for the entire network and the core network of more than 5 posts. The first finding is that, overall, the entire network is less polarized than the core network, as exemplified by the lower average polarity score. This means most of the active users are polarized; they will post predominantly on exclusively true information or misinformation threads. The second finding is that the average polarity score of the network decreased as the most influential users of the network were removed (the top 5% in the entire network and the top 10% in the core network). The 95% confidence intervals are generated from *t* tests testing the difference in means, with no overlap suggesting significance.

These results suggest several trends. The first is that the core network exhibits more echo chambering in its behavior. Thus, those that are more influential on PTT are more likely to be more polarized, gravitating toward true or false information. However, when interpreted in conjunction with the regression results, posts from polarized users were likely to arouse less attention, suggesting that polarized users are more active and influential in commenting instead of posting. The second is that polarization happens with a few key users. In the core network, when the top 10% of nodes using any metric are cut, the polarity score decreases sharply. After 10%, the rate of decrease tapers off. The steeper declines for outdegree and betweenness centrality suggest that identifying active commenters and brokers in the network may be more useful than identifying those that receive many comments.

## Discussion

### Principal Findings

This study aims to explore vaccine misinformation in Taiwan in 3 ways. The first is by cataloging the types of vaccine misinformation encountered during the pandemic. The second is comparing the diffusion of different types of misinformation. The third is measuring how influential users contribute to the level of polarity of the discussion forum. Overall, the results show that propaganda-style misinformation (RR=2.07; *P*<.05) has wider diffusion, whereas polarized users (RR=0.22; *P*<.001) received less attention for their posts. In addition, by removing the most influential users in the misinformation diffusion network, measured by indegree, outdegree, and betweenness centrality, the average polarity of the entire network decreased. This suggests a potential strategy to combating the echo chamber effect in misinformation by targeting the influential users.

The results from the regression supplement the literature on misinformation virality [53] that suggests that misinformation type may contribute to differences in diffusion. Misinformation propagated with political propaganda intentions has a higher rate of reposting compared to true information. This, in part, could be due to the nature of the PTT forum, which is a heavily politicized forum. In the broader echo chamber literature on science, there are growing concerns of politically aligned selective exposure in science. A study by Nisbet et al [54] focusing on the United States found that, regardless of political affiliation, there are negative and emotionally loaded reactions to dissonant science communication. The downstream effects of these trends are drastic in that overall trust of the scientific community diminishes. Some studies posit that this is due to a phenomenon of “elite cues” in which public opinion follows the elites’ partisan battles on the specific issue, such as shown in the climate change debate in the United States [55,56]. Another closer example to vaccination is Hamilton and Safford’s study [57] on trust in the US CDC among Republicans following Donald Trump’s changing views toward the CDC. Although this study does not analyze elite cues, the same trend may also be true for Taiwan, which has political systems very similar to the United States in terms of bipartisanship and polarization. A most recent cross-national study further suggests the close relationship between individuals’ voting choices and their conspiracy beliefs during the COVID-19 pandemic [58]. A further study on the narratives in the propaganda stream is one next step toward corroborating these trends in the literature, and enriching it by providing analysis from a different perspective.

Other than propaganda-based misinformation, the finding that disinformation was reposted less also reinforces that certain streams of misinformation may have more viral potential than others. On content, the findings corroborate other literature findings of longer posts being more transmissible. Posts with longer text are more likely to be spread [43]. However, more active users generally have proportionally less of their boards arouse attention, a finding different from other platforms like X [44]. This could be a natural trend for users whose post volumes are high.

Other findings similar to previous studies include the more prevalent engagement with misinformation compared to true information [59-62], as suggested by the skew of the graph in Figure 1A. This is true despite most boards containing true information. However, the diffusion of true information and misinformation is the same, as seen in Table 4. In addition, this study finds that most misinformation streams also tend to be in either vaccine hesitant or antivaccination stance boards [63-65], a finding not surprising given the affinity of misinformation to an antivaccination agenda [66]. Another new finding is that “brokers” of forum information—influential users as measured by betweenness—disproportionately engage with true information or misinformation across the entire network, suggesting that they are the polarizing forces in the echo chambering of misinformation. These results highlight the importance of measuring polarity in endorsement of misinformation for vaccine hesitancy, an aspect previously underexplored in the literature. By analyzing the polarized endorsement of misinformation, we gain insights into the formation of echo chambers of misinformation arising in a forum and can devise strategies for managing them. The findings for PTT suggest 2 main modes of moving forward in vaccine misinformation management for Taiwan.

The first is the implementation of a consolidated, automatic early detection system of online media potentially containing vaccine misinformation. Much like a disease surveillance system, a misinformation surveillance system should be able to detect and flag potential misinformation early such that its transmission is inhibited if necessary. The reason this is useful is because of the seeming affinity that misinformation has in attracting users, which is more dangerous when those users are influential. Once detected, this information can be used to inform the public as a means of “psychological inoculation” to help internet users better discern misinformation [67]. Given that the narratives of misinformation are not novel in Taiwan, this system can be trained using global reports of vaccine-related misinformation in addition to the civilian-led misinformation clarification platforms already present in Taiwan (MyGoPen, CoFacts, etc). This management system would be particularly important during outbreaks or other flashpoints as online information often peaks as a response to events [68-71]. As a step in infodemic management, it would help improve health literacy.

The second is to create a system that can identify then neutralize influential brokers (ie, high betweenness centrality) and commenters (ie, high outdegree) who have high interactions with misinformation posts. This can reduce the polarity in the network away from negative polarity. In this study, their connectivity means that neutralization may reduce the polarity of the network. Identifying users that are either antivaccination or spreaders of misinformation using these metrics represents an untapped potential for positive engagement that continually breaks the echo chamber effect for both vaccine stance and misinformation spread.

Although the technical aspects of such a system are relatively straightforward, the challenge extends beyond just technical regulation and public health, requiring the balancing of ethical considerations in moderating harmful information without impinging on free speech. This moral dilemma has been studied in other contexts, such as politics and culture [72,73], suggesting that the public supports misinformation management if it causes harm, defined as something undermining people’s ability to make informed decisions (particularly around public health and elections) [74]. Studies on the association of misinformation and vaccine intention suggest that they are negatively correlated [15,75]. Other considerations are the scope of required management, such as removal of posts or the temporary or permanent suspension of users. During the COVID-19 pandemic, many social media platforms (eg, Facebook, Instagram) assumed this regulatory role and intervened to prevent the spread of misinformation and conspiracies around vaccines. However, in Taiwan, no such action was taken to actively manage misinformation on local platforms. Rather, the government provided information platforms as secondary references for those already exposed. Moving forward, misinformation management should constitute part of the overall architecture for epidemic management in Taiwan, a process likely to involve fierce democratic discussion or debate on the moral dilemma of speech regulation.

### Limitations

A significant limitation of this study is its focus on a single platform, PTT, which may not be representative of the broader online landscape in Taiwan. PTT’s user base is distinct in its demographics and its heavily politicized nature, which could skew the findings on the spread of vaccine misinformation and its interaction with user behavior. As such, the results may not fully capture how misinformation diffuses across other popular platforms like Facebook, LINE, or Instagram, which have different user profiles and communication dynamics. Future research could address this limitation by conducting comparative studies across multiple social media platforms to gain a more comprehensive understanding of misinformation diffusion.

Another limitation stems from the use of social media data, which inherently introduces challenges related to user anonymity and the authenticity of user identities. Social media platforms often allow for pseudonymous accounts, making it difficult to verify whether the key users identified in this study are representative of broader population segments or if they include bots or automated accounts, which are known to play a role in the spread of misinformation. Additionally, social media data are typically unstructured, which presents challenges in accurately capturing the context, sentiment, and intent behind posts. This study relied on manual coding, which, despite high intercoder reliability, is subject to human bias. Incorporating more automated methods, such as natural language processing, could reduce bias and improve the scalability of the analysis. Moreover, the rapidly evolving nature of social media platforms, algorithms, and user behavior means that findings based on past data may not hold in future contexts, making longitudinal studies crucial for a more dynamic understanding of misinformation patterns over time.

### Conclusion

This study provides key insights into the dynamics of vaccine misinformation in Taiwan, highlighting the influence of core users in spreading polarizing content and the different diffusion patterns between true information and misinformation. The findings underscore the potential for targeted interventions, such as identifying and neutralizing influential users who propagate misinformation, as a means of reducing network polarization and improving public health outcomes. By addressing the mechanisms of misinformation diffusion and understanding the role of user behavior in its propagation, this research contributes to the broader effort to combat misinformation in digital spaces and enhance public trust in scientific communication. Future studies can build on these findings by extending the analysis to other platforms and exploring cross-national comparisons to generalize the results.

## Supplementary material

10.2196/57951 Multimedia Appendix 1 Testing different thresholds for the cutoff of the core group.

10.2196/57951 Multimedia Appendix 2 Correlation analysis for quantitative variables in negative binomial regression.

## Abbreviations

CDC: Centers for Disease Control and Prevention
RR: relative risk
