# Supplementary material for: The Role of Influencers and Echo Chambers in the Diffusion of Vaccine Misinformation: Opinion Mining in a Taiwanese Online Community
Source: JMIR Infodemiology. 2025 Aug 18;5:e57951. doi: 10.2196/57951 (PMC12360728; doi:10.2196/57951)

**Supplementary materials for**

**“Diffusion of vaccine misinformation in a Taiwanese online community: The role of influencers and echo chambers”**

Appendix 1:

S1. Testing different thresholds for the cutoff of core group (10 ≥ n ≥ 1, n=number of engagements in forum (comment, post))


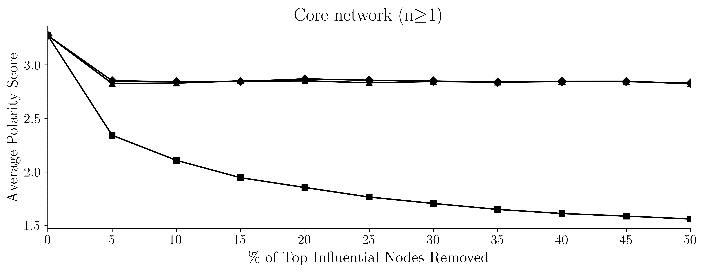

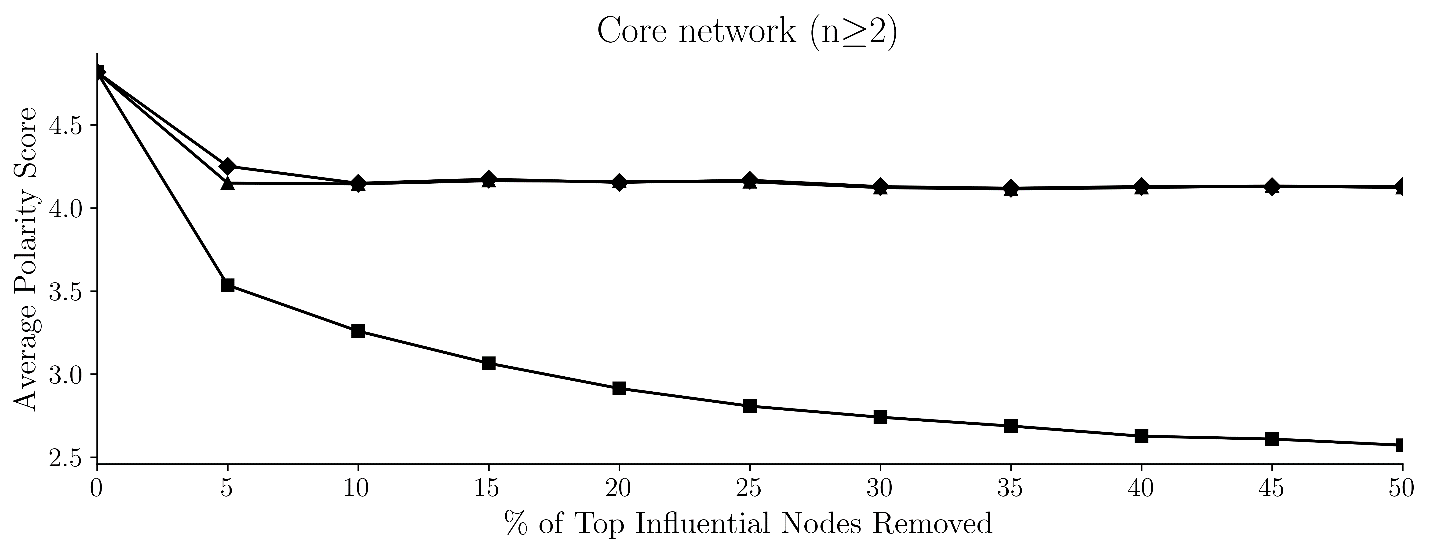


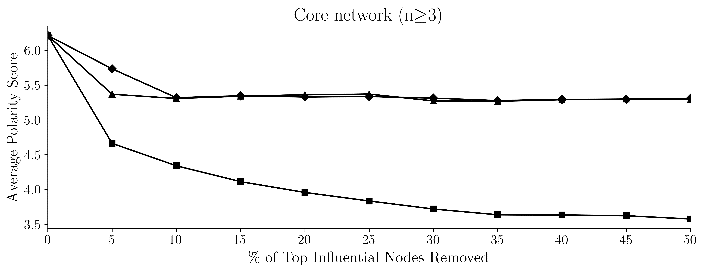

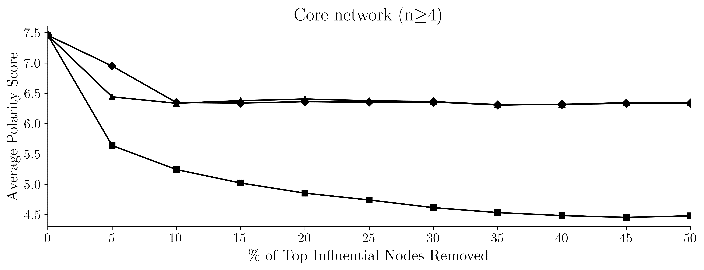


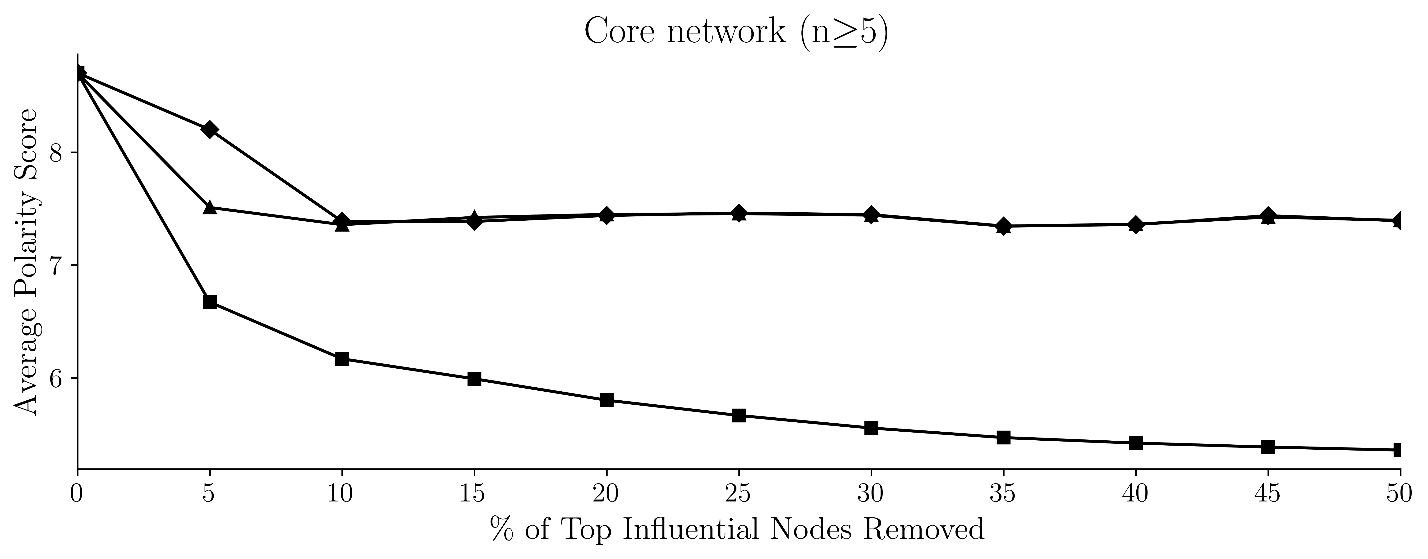

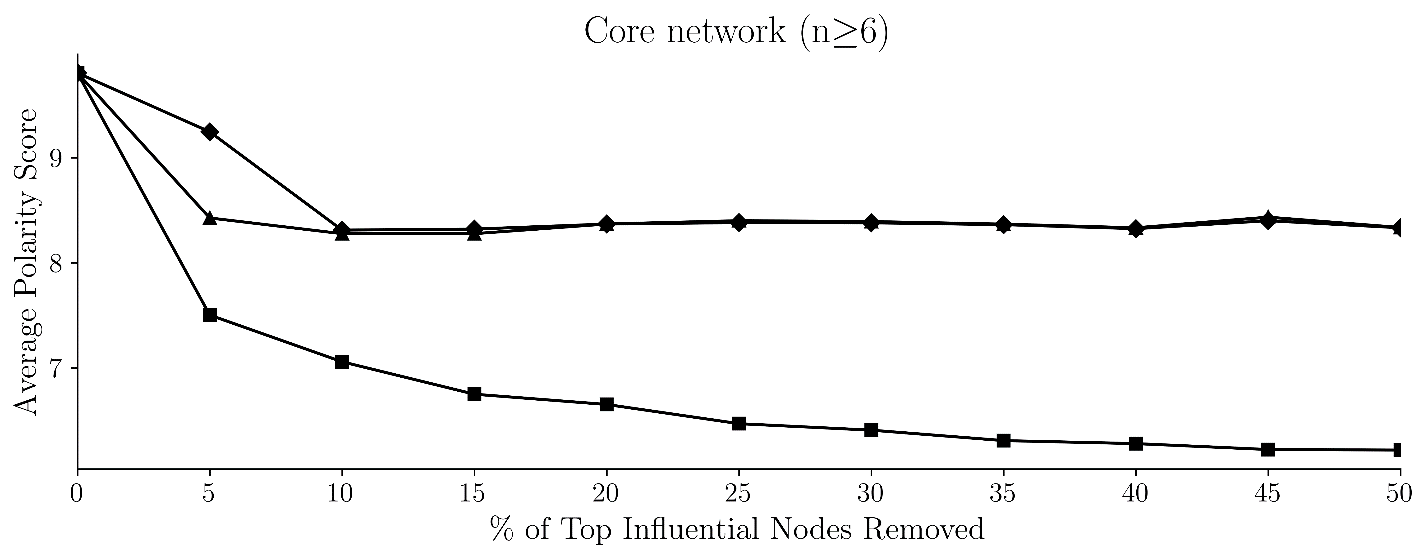


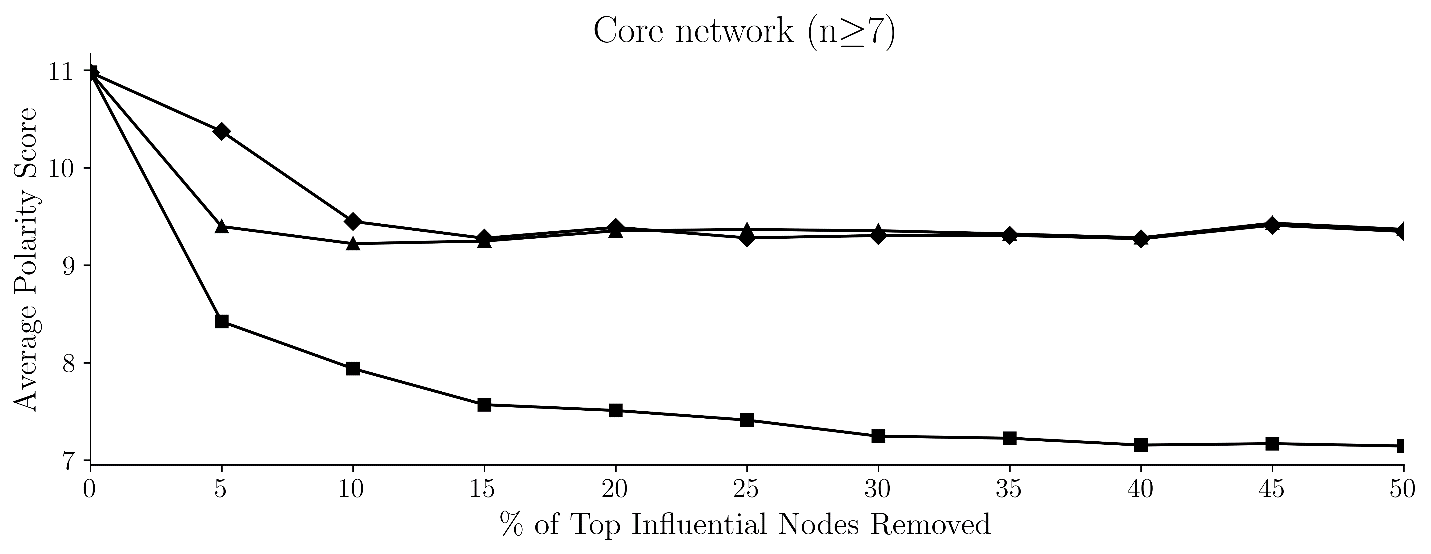

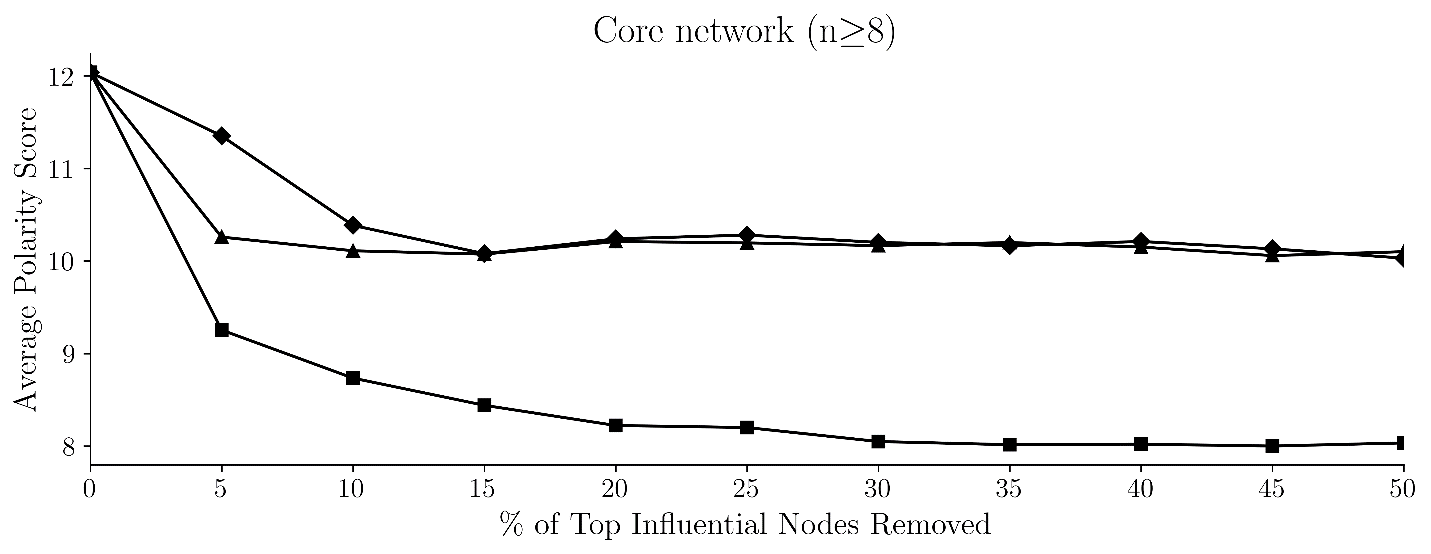


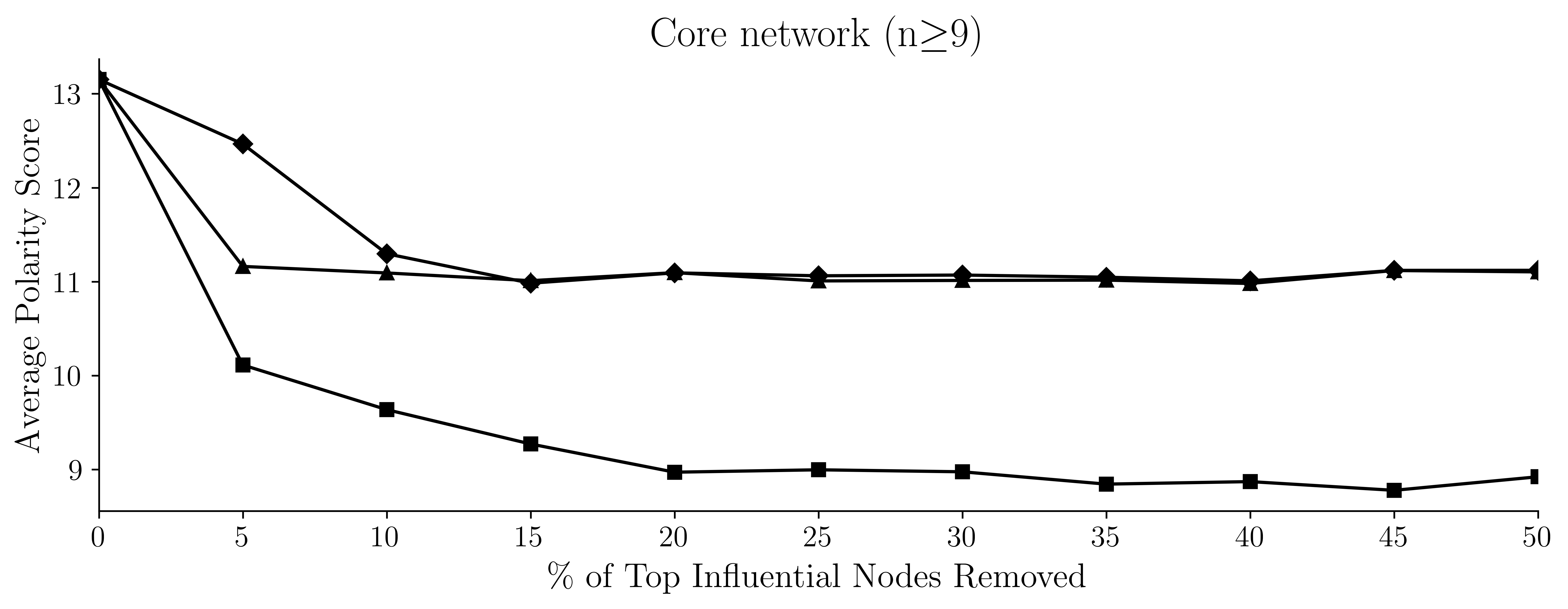

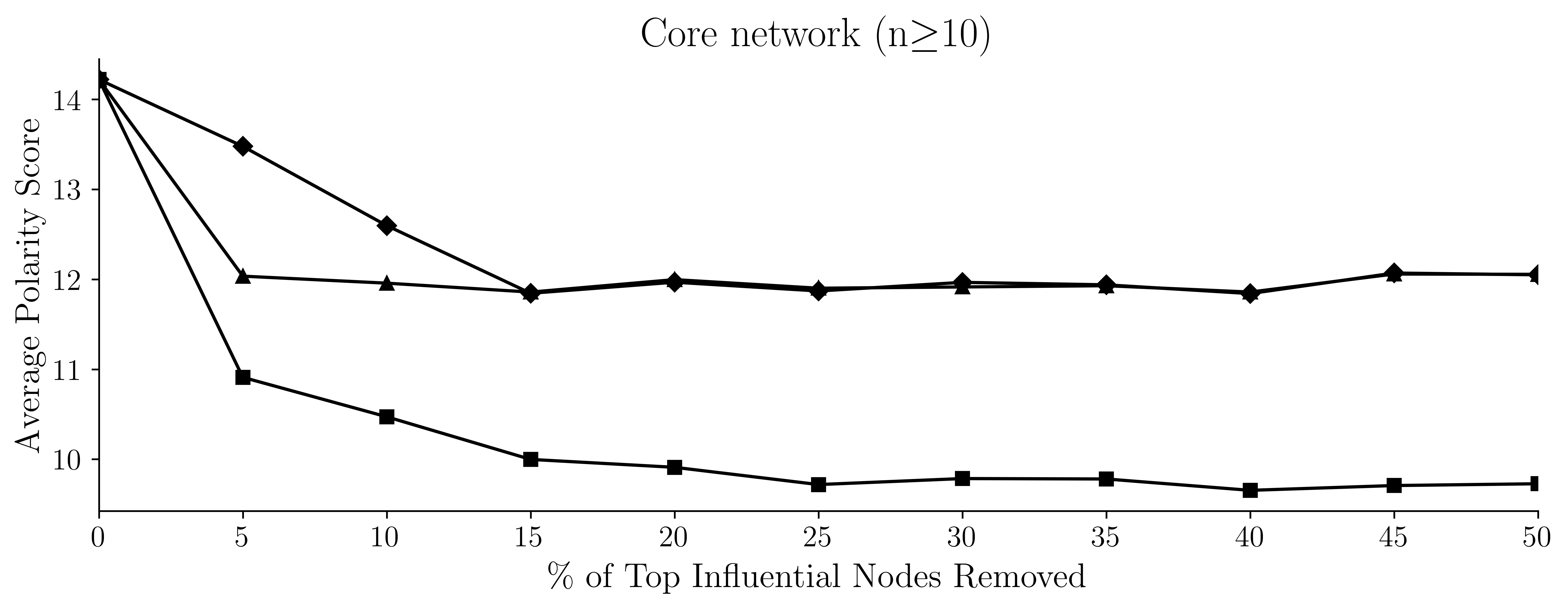

Supplement: Multimedia Appendix 1 [file infodemiology-v5-e57951-s001.docx]
