# Supplementary material for: The Role of Influencers and Echo Chambers in the Diffusion of Vaccine Misinformation: Opinion Mining in a Taiwanese Online Community
Source: JMIR Infodemiology. 2025 Aug 18;5:e57951. doi: 10.2196/57951 (PMC12360728; doi:10.2196/57951)
Supplement: Multimedia Appendix 2 [file infodemiology-v5-e57951-s002.docx]

**Supplementary materials for**

**“Diffusion of vaccine misinformation in a Taiwanese online community: The role of influencers and echo chambers”**

Appendix 2:

S2. Correlation analysis for quantitative variables in negative binomial regression

Table S2.1. Correlation of variables for breadth regression

|  | No. of reposts | Content length | Polarity (volume, absolute value) | User post count |
| --- | --- | --- | --- | --- |
| No. of reposts | 1.00 | 0.11 | -0.09 | -0.03 |
| Content length | 0.11 | 1.00 | 0.06 | -0.01 |
| Polarity (volume, absolute value) | -0.09 | 0.06 | 1.00 | 0.19 |
| User post count | -0.03 | -0.01 | 0.19 | 1.00 |


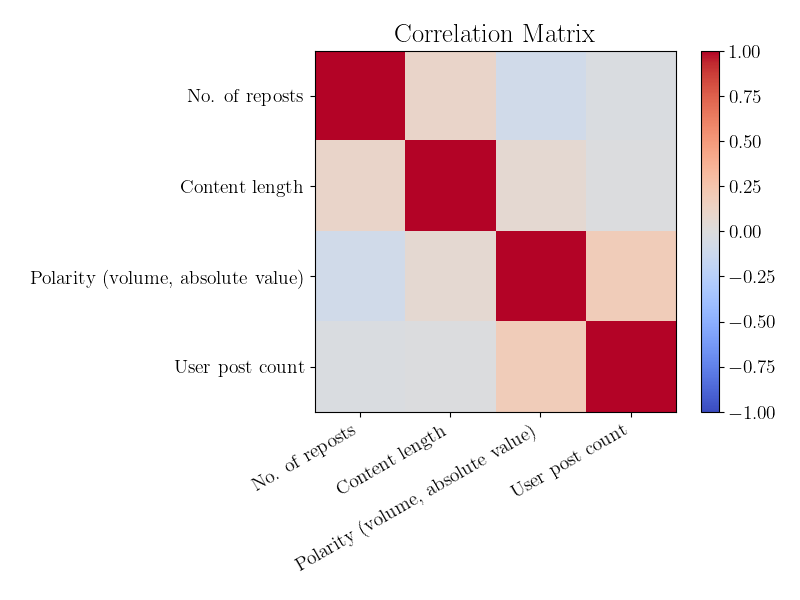


Figure S2.1 Correlation matrix of variables for breadth regression
